# Wide angle and narrow-band asymmetric absorption in visible and near-infrared regime through lossy Bragg stacks

**DOI:** 10.1038/srep27061

**Published:** 2016-06-02

**Authors:** Shiwei Shu, Yawen Zhan, Chris Lee, Jian Lu, Yang Yang Li

**Affiliations:** 1 Center Of Super-Diamond and Advanced Films (COSDAF), City University of Hong Kong, Tat Chee Avenue, Kowloon, Hong Kong; 2 Department of Physics and Materials Science, City University of Hong Kong, Tat Chee Avenue, Kowloon, Hong Kong; 3 City University of Hong Kong Shenzhen Research Institute, 8 Yuexing 1st Road, Nanshan District, Shenzhen, China; 4 Department of Mechanical and Biological Engineering, City University of Hong Kong, Tat Chee Avenue, Kowloon, Hong Kong

## Abstract

Absorber is an important component in various optical devices. Here we report a novel type of asymmetric absorber in the visible and near-infrared spectrum which is based on lossy Bragg stacks. The lossy Bragg stacks can achieve near-perfect absorption at one side and high reflection at the other within the narrow bands (several nm) of resonance wavelengths, whereas display almost identical absorption/reflection responses for the rest of the spectrum. Meanwhile, this interesting wavelength-selective asymmetric absorption behavior persists for wide angles, does not depend on polarization, and can be ascribed to the lossy characteristics of the Bragg stacks. Moreover, interesting Fano resonance with easily tailorable peak profiles can be realized using the lossy Bragg stacks.

Advanced absorbers are intensively studied for their importance in a wide range of applications, such as solar cells, photodetectors, sensors, nano-imaging devices, and thermal emitters^1^,^2^,^3^,^4^,^5^. The initial absorber design, targeting high absorption efficiencies for military applications in the microwave regime, was a bulky structure proposed by Salisbury in 1952^6^. In 2008, Landy *et al*.^2^ proposed a thin-film type of perfect absorber, opening a new route for perfect absorption using metamaterials^1^,^2^,^3^,^4^,^5^,^7^,^8^. The majority of these absorber designs focus on a triple-layered structure, which includes an elaborately patterned metallic top layer (e.g., an array of metal nano-disk), a thin (e.g., 20 nm) dielectric spacer layer, and a thick metal bottom layer^4^,^5^,^8^,^9^. Major efforts of absorber research have been devoted to minimize the angle-dependency and polarization-dependency, achieving encouraging progresses^3^,^9^,^10^,^11^,^12^,^13^,^14^,^15^,^16^,^17^,^18^,^19^.

Here in this study, in the first part, we propose a novel type of asymmetric absorber working in the visible and near-infrared region based on lossy Bragg stacks. The lossy Bragg absorbers are found to enable perfect absorption with wide angle tolerance and polarization insensitivity.

Conventional Bragg stacks are dielectric multilayers consisting alternating sublayers of high and low refractive indexes^20^,^21^. They can be regarded as a type of 1D photonic crystal^22^,^23^ with their working mechanism well studied. For their high reflection of the resonance wavelengths, dielectric Bragg stacks are utilized for wide ranging applications, such as vertical-cavity surface-emitting lasers (VCSEL)^24^,^25^,^26^, light-emitting diodes (LED)^27^,^28^,^29^, solar cells^30^, photodetectors^31^,^32^, Fabry-Perot filters^33^.

Although dielectric Bragg stacks have been intensively studied, little attention was paid to lossy ones historically. This is possibly due to the fact that Bragg stacks are commonly applied as reflectors and thus using absorptive lossy materials to construct Bragg stacks would be counter-intuitive. Interestingly, recent research has discovered new properties on lossy Bragg stacks made from metamaterials^34^,^35^, magnetoelectric materials^36^, and alternative metal/dielectric sublayers^37^,^38^, for super-resolution lens^37^, and asymmetric transmission^38^. For example, the fascinating asymmetric Bragg reflection in complex crystals has recently caught much attention within the context of passive parity-time symmetry^39^,^40^,^41^. Here in the second part of this study, we report the extraordinary narrow-band asymmetric absorption at and only at the resonance wavelengths of the lossy Bragg thin films, and reveal its possible mechanism. Furthermore, we show that these novel lossy Bragg thin films can be realized using multilayers made from porous metals (e.g., Ni or Pt) or metal-embedded dielectrics (e.g., Al-embedded Al_2_O_3_).

Finally, it needs to be pointed out that one major obstacle hindering the practical applications of advanced absorbers, particularly those in the visible and infrared region, arises from their elaborate nanostructured surface patterns, which not only lead to high fabrication cost but also limit the sample size. By contrast, the lossy Bragg thin films proposed here are free from elaborate surface patterns and can be conveniently fabricated over a large area using convenient vapor deposition or electrochemical methods^42^.

## Structural and theoretical model

**Figure 1:**
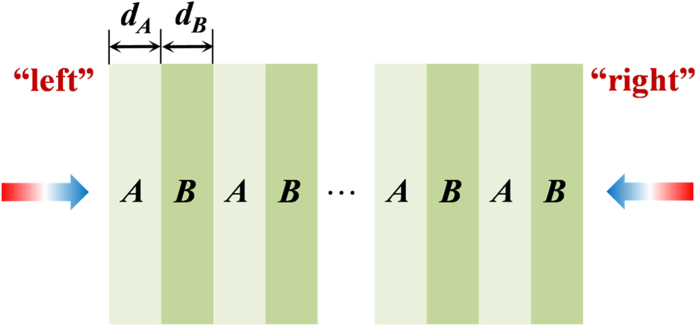
Schematic illustration of the proposed lossy Bragg stack.

The proposed lossy Bragg structure is schematically shown in Fig. 1, consisting alternating Sub-layers A and B, with the “left” surface being a Sub-layer A and the “right” surface being a Sub-layer B. The light propagation directions are also shown in Fig. 1: incident from “left” – light incident on a surface sub-layer of A; incident from “right” – light incident on a surface sub-layer of B. The thicknesses of the sub-layers A and B are denoted as *d*_*A*_ and *d*_*B*_. The complex refractive index of the sub-layers A and B are described as *ñ*_*A*_ = *n*_*A*_ + *κ*_*A*_*i* and *ñ*_*B*_ = *n*_*B*_ + *κ*_*B*_*i*, while the relative permeability of sub-layers A and B is regarded as 1. The period number (i.e., the repeating number of AB or BA in the structure) of the Bragg stack is denoted as *m*. For simplicity, we set *k*_*A*_*d*_*A*_ = *k*_*B*_*d*_*B*_, where *k*_i_ = *ñ*_*i*_(ω/c) (i = A, B). At resonance wavelengths,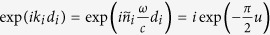, where 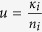. In this paper, we used the Transfer Matrix method (TMM) and the finite-difference time-domain (FDTD) method^43^ to calculate the optical properties of lossy bragg stacks.

## Results and Discussion

**Figure 2:**
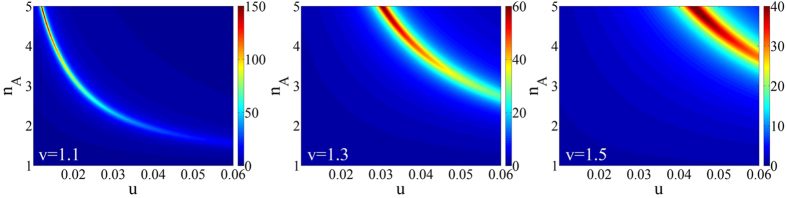
*DoA* (the degree of asymmetry) dependence on *u* and *n*_*A*_, when *v* = 1.1, 1.3, and 1.5.

It is set that 
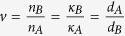
 and 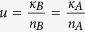. Note that the ratio between the real and imaginary parts of the refraction indexes is set to be the same here is for the calculation convenience, but not a necessity. The TMM produces the complex wavelength-dependent parameters reflectivity, *r*, and transmissivity, *t*. Reflection, transmission and absorption are defined as 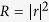, 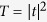, and *A* = 1 − *R* − *T*, respectively. It is assumed that the lossy Bragg structure is thick enough to make T equal to near zero, for simplicity, we assumed *A* = 1 − *R*, indicating the equivalence of the reflection asymmetry and the absorption asymmetry. Figure 2 shows that the reflection asymmetry depends on the parameters *n*_*A*_, *v* and *u*. The color scale in Fig. 2 represents the degree of asymmetry (*DoA*) which is defined as *DoA* = *R*_*right*_/*R*_*left*_. When *v* is increased, the maximum value of *DoA* is decreased. Meanwhile, this maximum tends to appear at high value of *n*_*A*_. The maximum value of *DoA* was found when *n*_*A*_ = 4.9, *u* = 0.01, *v* = 1.08. Unless otherwise stated, the default parameters are set as follows: *ñ*_*A*_ = 4.9 + 0.049 i, *ñ*_*B* _= 5.3 + 0.053 i, *m* = *30, d*_*A* _= 25.5 nm, and *d*_*B* _= 23.6 nm.

**Figure 3:**
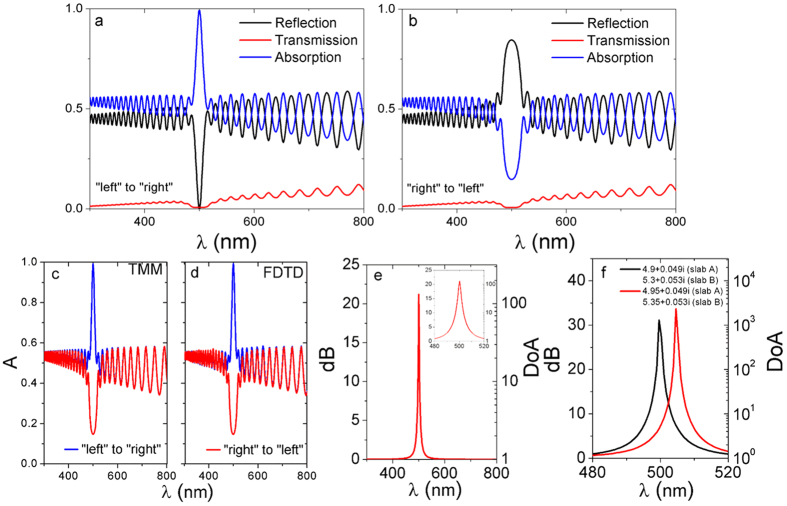
Reflection, Transmission and Absorption spectra of typical lossy Bragg stack when light propagate from “left” to “right” (**a**) and from “right” to “left” (**b**). Absorption spectra of the typical lossy Bragg stack simulated using the TMM (**c**) and FDTD method (**d**). The degree of asymmetry (*DoA*) and its *dB* value [*dB* = 10lg(*DoA*)] calculated using the TMM are shown in (**e**). The *DoA* peak appears to be sensitive to the refractive indexes of the sub-layers. The spectra in (**f** ) shows the shift of the *DoA* peak when *ñ*_*A*_ and *ñ*_*B*_ are changed from 4.90 + 0.049 i and 5.30 + 0.053 i (“original”) to 4.95 + 0.049 i and 5.35 + 0.053 i (“after change”), respectively.

The reflection, transmission and absorption spectra of typical lossy Bragg stack were shown in Fig. 3(a,b) for different light propagation directions. The finite-difference time-domain (FDTD) method was also adopted to calculate the absorption spectra. For FDTD simulation, we have used periodic boundary conditions along the x and y axes, and PML boundary conditions along z axis (the light propagation direction). Unlike otherwise stated, the structural periodicity of the lossy Bragg stack is set as 30. The absorption spectra of a lossy Bragg structure calculated using the TMM and FDTD method are shown in Fig. 3(c,d). At the Bragg resonance wavelength of 500 nm, an absorption peak and dip are observed for light propagating from “left” and from “right”, respectively (see Fig. 1 for the “left” and “right” sides). It should be pointed out that the Bragg resonance wavelength can be easily controlled by adjusting the structural or optical parameters of the Bragg stack, e.g., by tuning the thickness of the sub-layers. Notably, little absorption/reflection asymmetry is observed for the rest of the spectra. Therefore, a novel thin-film type of optical device whose two surfaces are able to exhibit dramatically different optical response selectively only within an exceptionally narrow band of several nanometers Fig. 3(d) in the visible and near-infrared spectrum can be achieved using the design proposed in this study. Furthermore, the *DoA* peak appears to be sensitive to the changes of the refractive index, indicating the potential optical sensing applications of the lossy Bragg structures. For example, when *ñ*_*A*_ is changed from 4.90 + 0.049 i to 4.95 + 0.049 i, and *ñ*_*B*_ changed from 5.30 + 0.053 i to 5.35 + 0.053 i, a notable red-shift of the *DoA* peak is resulted in Fig. 3(e). Considering the high similarity of the mathematic models between the lossy and dielectric Bragg stacks, the tolerance of the optical responses of the proposed lossy Bragg stacks to the structural or optical parameter imperfection should be at a very similar level as the conventional dielectric Bragg stacks.

One may ask why the lossy Bragg structure displays the above-discussed absorption asymmetry at the resonance wavelength, whereas the dielectric Bragg structure does not. To explain this discrepancy, let us consider one period first. Its corresponding transfer matrix can be written as^44^:


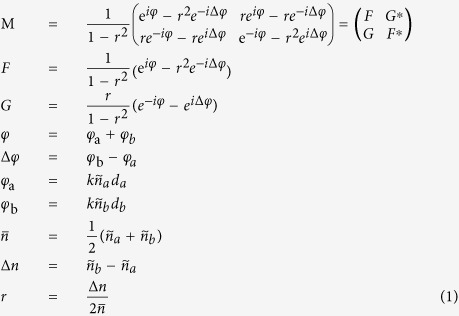


For a Bragg stack with the number of periodicity, *m*, the corresponding transfer matrix can then be written as:


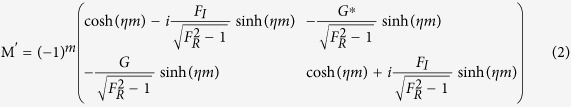


where *F*_R_ is the real part of *F, F*_I_ the imaginary part of *F*, and *η* is piecewise defined as


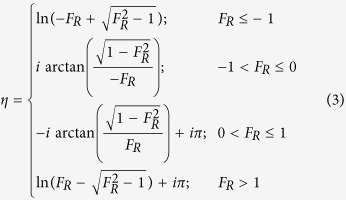


The respective reflection for forward and backward directions is:


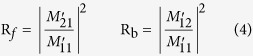


It is obvious that the difference between the forward and backward reflection is correlated with the difference between *G* and G^*^.


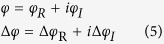



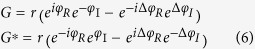


From above equations, the reflections (*R*_*f*_ *and R*_*b*_) are equal for dielectric structures (*u* = 0). However, when lossy material is used (*u* ≠ 0), the models of *G* and G* are different, leading to reflection asymmetry. It should be pointed out that, by carefully designing the optical parameters of the lossy Bragg stack proposed in this study, it is potentially feasible to accomplish the recently reported asymmetric Bragg reflection that was realized in the complex crystals and PT-symmetric periodic structures^39^,^40^,^41^.

**Figure 4:**
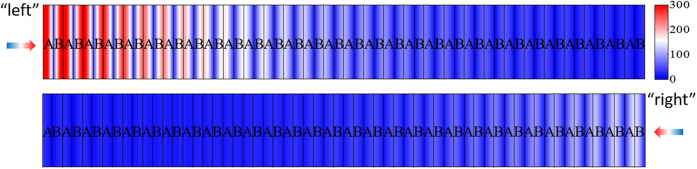
Electric field distribution (at *λ* = 500 nm) of the typical lossy Bragg stack for light propagating from “left” to “right” (upper) and from “right” to “left” (lower).

The electric field distribution (at λ = 500 nm) for the lossy Bragg stack was simulated using the FDTD method (Fig. 4). When propagating from “left”, most light enters the structure and gets absorbed; On the contrary, most light is reflected when propagating from “right”.

**Figure 5:**
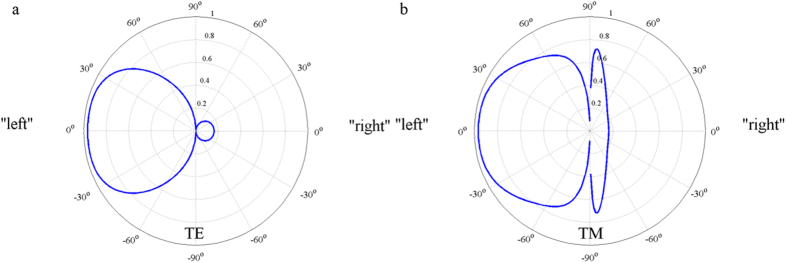
Absorption radar diagram of the typical lossy Bragg structure for the TE and TM polarizations at *λ* = 500 nm with light propagating from two directions.

The asymmetric absorption/reflection behaviors of the lossy Bragg structures are found insensitive to TE polarization (Fig. 5a) and TM polarization (Fig. 5b) and possess good tolerance to wide incident angles. When light propagating from “left”, an absorption efficiency of 50% is observed at the incident angle of 60° and 85° for the TE and TM polarization, respectively. For light propagating from “right”, low absorption (high reflection) is sustained up to 90° for the TE polarization, while for the TM polarization low absorption is observed up to 60°. The high absorption emerges at high angles (~82°) for the TM polarization can be explained by the Brewster’s law.

**Figure 6:**
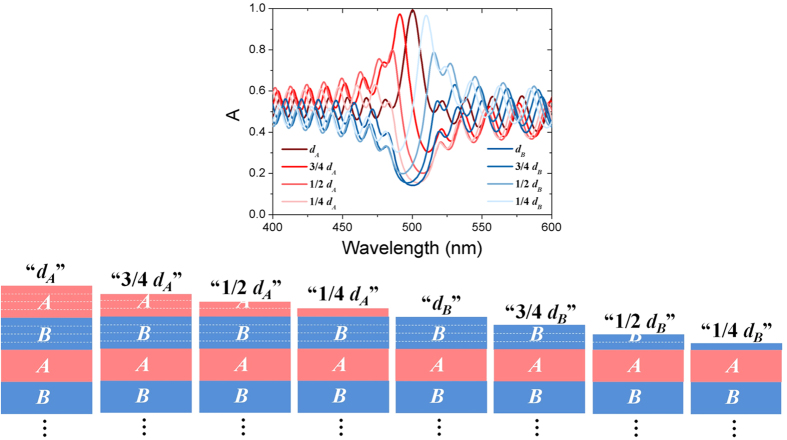
Absorption spectra with different Fano profile of the lossy Bragg stack with the corresponding top sub-layer thickness marked in the legend. The spectra were simulated using the TMM. The drawing illustrates the corresponding structures.

Interestingly, by changing the thickness of the top Sub-layer in the lossy Bragg stack, the profile of the resonance absorption/reflection spectrum can be varied precisely from symmetric to asymmetric (Fig. 6), i.e., the Fano resonance can be induced. Remarkably, by adjusting the thickness of the top sub-layer of the lossy Bragg structure, the peak profile can be rotated from 0° to 360° continuously (Fig. 6). The Fano resonance observed here is possibly resulted from the interference between the radiative mode and the localized mode (Tamm states). The radiative mode originates from the periodic multilayered structures. Previous studies^45^,^46^ have shown that the Tamm states are localized at the interface between a truncated periodic multilayer and a semi-infinite homogeneous medium (e.g., air). Here in this study, when the surface layer (taking Sublayer A for example) of a lossy Bragg stack is truncated, the wavevector of Tamm states for TE and TM polarizations can be respectively written as^45^:


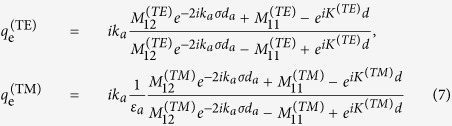


Here, *k*_*a*_ is the wavevector in Sub-layer A, *M*_*11*_ and *M*_*12*_ the elements of the transfer matrix of one period, 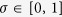 the thickness portion of the truncated Sub-layer A, *K* the Bloch wave vector, *d* the thickness of one period, and *d*_*a*_ the length of Sub-layer A. The Tamm states (localized mode) exist when and only when there is a truncated surface sublayer, so do the Fano-type profiles in the absorption spectra, confirming that the presence of Fano resonance is due to interference between the localized mode (Tamm states) and the radiative mode (originated from periodic structures).

For practical applications, porous metals (e.g. Pt, Ni, Pd, Cr) can be used to construct the proposed lossy Bragg structures. Regarding the manufacturing method, the lossy Bragg structures can be potentially constructed by the convenient vapor deposition or electrochemical method. The Lorentz-Drude (LD) model was used to describe metals with the relative complex permittivity^47^,^48^ (the relative permeability is 1). The effective permittivity of the porous metal sub-layers can be tuned by controlling its porosity. For example, let us set the porosities of Sub-layers A and B, *p*_*A*_ and *p*_*B*_, 0.4 and 0.3, respectively (i.e. the metal volume percentage of Sub-layers A and B, fa and fb,is 0.6 and 0.7, respectively), the thickness of each sub-layer 100 nm, and the periodicity 15. In the condition of the porous metallic feature sizes of the porous sub-layers significantly smaller than the light wavelength, each sub-layer can be treated as a homogeneous material with the effective permittivity, *ε*_*eff*_. According to the Maxwell-Garnett effective medium theory (MGEMT),


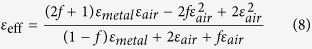


**Figure 7:**
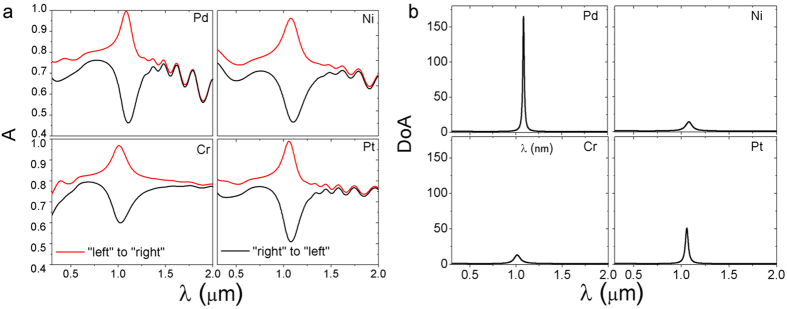
Absorption spectra (**a**) and *DoA* (**b**) of the lossy Bragg stacks based on Pd, Ni, Cr, and Pt (*p*_*A*_ = 0.4, *p*_*B*_ = 0.3, *d*_*A*_ = *d*_*B*_ = 100 nm, and *m* = 15).

The absorption behaviors of metal-based lossy Bragg stacks are simulated (Fig. 7a). Among the different metals simulated (Pd, Ni, Cr, and Pt), Pd shows the most dramatic absorption/reflection asymmetry with highest value of *DoA* (Fig. 7b). The porous Pd-based Bragg stack displays a near-perfect (99.6%) absorption peak for light propagating from “left”, and an absorption dip (45% efficiency) for light propagating from the opposite direction.

**Figure 8:**
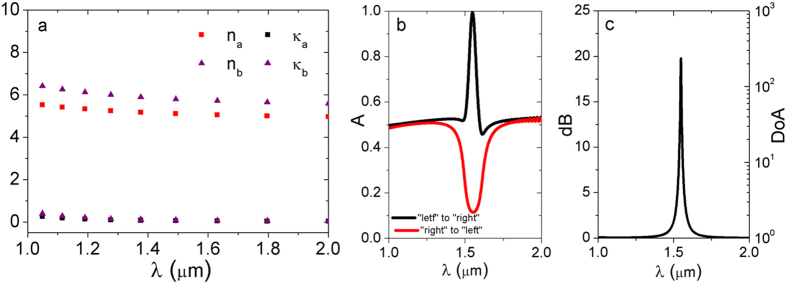
For the lossy Bragg stack made from Al-embedded Al_2_O_3_ (Al vol% = 70%, 75% and sublayer thickness = 73.5, 70 nm for Sublayers A and B, respectively, refractive index of Al_2_O_3_ = 1.76, and period number = 100): (**a**) real part and imaginary part of the effective complex refractive index for Sublayers A and B; (**b**) absorption spectra simulated using the TMM; (**c**) Degree of Asymmetry (*DoA*) and its *dB* value [*dB* = 10lg(*DoA*)] calculated using the TMM.

In order to enhance the relatively low *DoA* value observed for pure metal-based lossy Bragg stacks, other material systems are further investigated. Encouragingly, for practical applications at the telecom wavelength of 1550 nm, the composite material of Al nanoparticles-embedded Al_2_O_3_ has been identified as a promising candidate for constructing the proposed lossy Bragg stacks. Experimentally, the Al/Al_2_O_3_ based Bragg stacks can be constructed through either the convenient vapor deposition (e.g., by co-sputtering Al and Al_2_O_3_) or electrochemical (e.g., by electrodepositing Al in the anodic Al_2_O_3_ template) methods. When the Al particle sizes are significantly smaller than the light wavelength, each sub-layer can be treated as a homogeneous material. The Lorentz-Drude (LD) model was used to describe metals with the relative complex permittivity (the relative permeability is 1). The effective permittivity (*ε*_*eff*_) of Sub-layer A or B can be tuned by adjusting the Al volume percentage (*f*) in the corresponding sublayer (Fig. 8a). According to the Maxwell-Garnett effective medium theory (MGEMT),


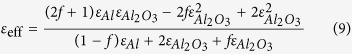


when the refractive index of Al_2_O_3 _= 1.76, Al vol% = 70% for Sublayer A, Al vol% = 75% for Sublayer B, *d*_*A*_ = 73.5 nm, *d*_*B*_ = 70 nm, and *m* = 100, the desired asymmetric absorption behaviors are clearly observed (Fig. 8b) with a sharp *DoA* peak located at the resonance wavelength of 1550 nm (Fig. 8c).

## Conclusion

Lossy Bragg structures have been theoretically investigated as a novel type of asymmetric absorber/reflector in a narrow bandwidth, insensitive to the incident angle and polarization. The reflection coefficient of the lossy Bragg stacks has been derived by the Transfer Matrix method, revealing that the asymmetry is mainly due to the lossy characteristics of the stacks. Interestingly, Fano resonance can be potentially realized using the lossy Bragg structures as well. For realistic device construction, Al embedded Al_2_O_3_ can be used to construct the proposed lossy Bragg structures. Furthermore, According to the Kirchhoff’s law^49^, the proposed Bragg absorbers may also be used as asymmetric thermal emitters, enabling greatly improved emitter performance by selectively suppressing emission from the non-active surface. Moreover, the novel metal-based Bragg absorbers can fully utilize the many desirable attributes of metals, (e.g., high conductivities), and enable novel multi-functional optical devices for sensing, logic gates, unidirectional thermal emission, and photovoltaic cells. For example, ultra-sensitive optical sensors, or photocatalytic thin-film electrodes with the two surfaces having dramatically different catalytic efficiencies at targeted wavelengths, can be realized. Another potential photovoltaic application based on the Thomson effect involves irradiating the lossy Bragg thin films to produce a temperature gradient and further induce a voltage across the two film surfaces. Furthermore, the concepts and structural designs proposed in this study may potentially be applied in phononic crystals, to realize wavelength-selective asymmetric phonon responses^50^.

## Additional Information

**How to cite this article**: Shu, S. *et al*. Wide angle and narrow-band asymmetric absorption in visible and near-infrared regime through lossy Bragg stacks. *Sci. Rep.* **6**, 27061; doi: 10.1038/srep27061 (2016).
